# From Lysosomal Storage Diseases to NKT Cell Activation and Back

**DOI:** 10.3390/ijms18030502

**Published:** 2017-02-25

**Authors:** Cátia S. Pereira, Helena Ribeiro, M. Fatima Macedo

**Affiliations:** 1 Instituto de Investigação e Inovação em Saúde, Universidade do Porto, Rua Alfredo Allen, 208, 4200-135 Porto, Portugal; cspereira@ibmc.up.pt (C.S.P.); helena.ribeiro@ibmc.up.pt (H.R.); 2 IBMC–Instituto de Biologia Molecular e Celular, Universidade do Porto, Rua Alfredo Allen, 208, 4200-135 Porto, Portugal; 3 Departamento de Química, Universidade de Aveiro, Campus Universitário de Santiago, 3810-193 Aveiro, Portugal; 4 Departamento de Ciências Médicas, Universidade de Aveiro, Campus Universitário de Santiago Agra do crasto-edifício 30, 3810-193 Aveiro, Portugal

**Keywords:** NKT cells, Lysosomal storage diseases, CD1d, lipids, lysosome

## Abstract

Lysosomal storage diseases (LSDs) are inherited metabolic disorders characterized by the accumulation of different types of substrates in the lysosome. With a multisystemic involvement, LSDs often present a very broad clinical spectrum. In many LSDs, alterations of the immune system were described. Special emphasis was given to Natural Killer T (NKT) cells, a population of lipid-specific T cells that is activated by lipid antigens bound to CD1d (cluster of differentiation 1 d) molecules at the surface of antigen-presenting cells. These cells have important functions in cancer, infection, and autoimmunity and were altered in a variety of LSDs’ mouse models. In some cases, the observed decrease was attributed to defects in either lipid antigen availability, trafficking, processing, or loading in CD1d. Here, we review the current knowledge about NKT cells in the context of LSDs, including the alterations detected, the proposed mechanisms to explain these defects, and the relevance of these findings for disease pathology. Furthermore, the effect of enzyme replacement therapy on NKT cells is also discussed.

## 1. Introduction

The lysosome, designated as the recycling compartment of the cell, was initially described by Christian de Duve in 1955 [1]. It is a membrane-enclosed organelle, characterized by its acidic pH and the presence of a large number of hydrolases. Genetic defects in lysosomal hydrolases or in other proteins necessary for the degradation or transport of macromolecules in the lysosome lead to lysosomal storage diseases (LSDs). The main feature of LSDs is the accumulation of different types of molecules in the lysosome, leading to a disturbance in lysosomal homeostasis that has important implications in autophagy, protein degradation, and metabolic stress [2,3]. The most usual classification of LSDs is based on the type of material that is accumulated. LSDs are divided in sphingolipidoses (accumulation of sphingolipids), mucopolysaccharidoses (accumulation of glycosaminoglycans), mucolipidoses (accumulation of glycolipids, glycosaminoglycans, and oligosaccharides), and glycoproteinoses (accumulation of glycoproteins) [4]. The most common LSDs are sphingolipidoses, which are usually characterized by the accumulation of glycosphingolipids (GSLs): ceramide or sphingosine molecules modified by the addition of sugar head groups. GSLs have been implicated in important immunological processes, such as T cell activation. More specifically, GSLs were shown to be antigenic for Natural Killer T (NKT) cells, a group of lipid-specific T lymphocytes with important functions in autoimmunity, infection, and cancer [5].

## 2. NKT Cells

NKT cells comprise a population of T lymphocytes with lipid-specific T cell receptors (TCRs). Peptide-specific T cells recognize antigens bound to Major Histocompatibility Complex (MHC) molecules at the surface of antigen presenting cells. Instead, NKT cells recognize lipid antigens that are bound to CD1d. CD1d stands for cluster of differentiation 1 d. In humans; CD1d molecules belong to a family of 5 MHC-class I like glycoproteins with hydrophobic grooves that have affinity for lipids. They are divided into three groups. Group I includes CD1a, CD1b, and CD1c isoforms. Group II includes CD1d, and group III is composed of CD1e. Group I and group II CD1 molecules present lipid antigens to lipid-specific T cells, while CD1e has a role in the loading of lipids in other CD1 molecules. Importantly, all these molecules traffic through the endo-lysosomal compartments and therefore are likely to be affected in LSDs. This review focuses on CD1d-restricted T cells, the NKT cells, the most studied lipid-specific T cells [6].

### 2.1. Classification and Characterization

Two different populations of NKT cells can be distinguished based on the TCR that they express (Table 1). Type I NKT cells, or invariant NKT (iNKT) cells, are characterized by the expression of a semi-invariant TCR composed of a Vα24Jα18 chain and a Vβ11 chain in humans, or a Vα14Jα18 chain paired with a limited repertoire of Vβ chains in mice [7,8,9,10].

On the contrary, type II NKT cells express variable TCRs. However, both mouse and human type II NKT cells present a bias towards some Vα and Vβ chains, suggesting that this population has some degree of oligoclonality [9,10]. These differences in TCR expression result in distinct antigen specificities (Table 1). While iNKT cells present a preference for α-linked monohexosylceramides, most known antigens for type II NKT cells are β-linked GSLs or phospholipids [11,12,13,14,15,16]. At the moment, there are no cell surface markers that allow for the identification of all type II NKT cells. However, iNKT cells can be easily recognized by the use of antibodies against a specific region of the semi-invariant TCR (6B11 antibody) or by CD1d tetramers loaded with the antigen α-galactosylceramide (α-GalCer) or analogues [17,18] (Table 1). The use of antibodies against only Vα24 or Vβ11 is not recommended for iNKT cell identification, as this will also detect T cells that are not restricted to CD1d [19].

#### 2.1.1. iNKT Cells

iNKT cells are abundant in adipose tissue, and influence the development of obesity and metabolic disorder [20,21,22,23,24,25,26]. They are also frequent in the mouse liver and present, although at lower amounts, in the spleen, peripheral blood, lymph nodes, bone marrow, and thymus [19]. The development of iNKT cells in the thymus requires the interaction of thymocytes expressing the semi-invariant TCR with CD1d molecules loaded with self-antigen and expressed in other thymocytes [27]. Once outside the thymus, iNKT cells are able to respond very rapidly to both TCR-mediated and TCR-independent activation, producing large amounts of a broad array of both anti-inflammatory and pro-inflammatory cytokines [28]. In humans, iNKT cells can be divided into functionally different subsets, according to the cell surface expression of CD4 and CD8 molecules. iNKT cells that do not express CD4 (which include both CD8+ and CD8− cells) are characterized by the production of similar amounts of pro-inflammatory and anti-inflammatory cytokines [18,29,30,31]. Importantly, CD8 expression is associated with an increase in cytolytic activity by iNKT cells, as well as an increase in the production of pro-inflammatory cytokines when compared to anti-inflammatory cytokines [31]. On the other hand, cells expressing CD4 display lower cytolytic activity and tend to produce larger amounts of anti-inflammatory cytokines and lower quantities of pro-inflammatory cytokines [18,29,30,31]. The expression of the NK cell marker CD161 has also been associated with a functional subset of iNKT cells that produce IL (interleukin)-17 in response to pro-inflammatory conditions [32]. In mice, iNKT cells were shown to have plasticity. Under certain conditions, peripheral iNKT cells can acquire the ability to produce IL-17 and IL-9, despite being committed to other functions when they leave the thymus [33,34].

#### 2.1.2. Type II NKT Cells

The identification of type II NKT cells is difficult to achieve due to the lack of specific surface markers and the diversity of CD1d-loaded lipids they recognize, thereby removing the possibility of using CD1d-tetramers to identify the entire type II population. Type II NKT cells can be identified by their CD1d-restriction and the absence of the invariant Vα14/Vα24 TCR α-chain. Type II NKT cells were initially described by comparing MHC-deficient mice (lacking conventional T cells) with MHC/CD1d double knockouts (lacking both conventional T cells and NKT cells). These studies identified a population of T cells in the MHC-deficient mice that did not recognize α-GalCer nor expressed the invariant TCR [35,36]. Later studies analyzing type II NKT cells were done by comparing mice lacking iNKT cells (Jα18-deficient mice) with mice lacking both iNKT cells and type II NKT cells (CD1d-deficient mice). These animal models have been very useful in defining the unique role of type II NKT cells in several pathological conditions including cancer, infection, and autoimmunity (the function of type II NKT cells has been extensively reviewed elsewhere [9,10]).

In humans, type II NKT cells are very frequent in bone marrow and liver [37,38]. However, in previous studies, type II NKT cells were identified as being positive for CD3 and CD56 or CD161, and negative for Vα24Jα18 and Vβ11 chains. Therefore, some non-CD1d-restricted T cells might have also been detected. Studies using CD1d tetramers loaded with specific lipids suggest that type II NKT cells outnumber iNKT cells in peripheral blood because frequencies of type II NKT cells specific for just one antigen are similar or greater for type II NKT than for iNKT cells [13,39,40]. The study of lipid-specific type II NKT cells with CD1d tetramers has also revealed other phenotypic and functional characteristics of type II NKT cells. CD1d tetramers loaded with sulfatide identified the majority of peripheral blood sulfatide-reactive NKT cells as γδ T cells, which expressed the Vδ1 segment [40]. Another study using CD1d dimers loaded with lysophosphatidylcholine identified a group of αβ CD3+ cells, negative for the Vα24Jα18 and Vβ11 chains, in the peripheral blood of control subjects and multiple myeloma patients [39]. These cells can express CD4, CD8, or none of these molecules. Functionally, they produce several cytokines upon stimulation, including IL-13, IL-5, IL-8, and IFN-γ [39]. More recently, CD1d tetramers loaded with β-glucosylsphingosine and β-glucosylceramide identified a subset of type II NKT cells, designated NKT follicular-helper cells, that provide direct help to B cells [13].

### 2.2. The Role of the Lysosome in NKT Cell Biology

Most of the studies that addressed the role of lysosomes in CD1d-mediated lipid antigen presentation focused only on iNKT cells. However, it is expected that some of the described mechanisms are also applicable for type II NKT cells.

There are mainly three lysosomal characteristics that contribute to the role of this organelle in iNKT cell activation: the content of hydrolytic enzymes capable of processing lipid antigens specific to iNKT cells; the content of lipid-transfer proteins (LTPs) that assist lipid loading in CD1 molecules; and the co-localization of CD1d molecules with lipid antigens in a low-pH compartment.

The processing of glycolipids by lysosomal hydrolases can lead both to the generation or inactivation of iNKT cell antigens. The role of lysosomal enzymes on the generation of lipid antigens was initially demonstrated for a synthetic lipid, Gal(α1→2)-α-galactosylceramide, which upon galactose removal by α-galactosidase A (the enzyme deficient in Fabry disease), becomes an iNKT cell antigen [41]. However, it was suggested that naturally-occurring glycolipids, such as α-galactopyranosyl(1→2)-α-glucosyl diacylglycerol from *Streptococcus pneumoniae*, might also need galactose removal to become antigenic [42]. Studies with Sandhoff disease mice showed that β-hexosaminidase activity was important for removing β-linked *N*-acetylgalactosamine from isoglobotetrahexosylceramide, yielding the iNKT cell antigen isoglobotrihexosylceramide [43]. Lysosomal phospholipase A2, responsible for the generation of lysophospholipids from phospholipids, was shown to be required for the normal presentation of endogenous antigens to iNKT cells [44,45]. However, the specific endogenous antigens processed by this enzyme were not identified [45]. Recently, lysosomal enzymes were also shown to be involved in the degradation of iNKT cell antigens through a sequential process that requires acid ceramidase (defective in Farber disease) to remove the acyl chain, and α-galactosidase A to remove the galactose residue [46].

Inside cells, lipids usually travel in membranes or in association with proteins. Lysosomal LTPs facilitate loading in CD1d molecules, either by promoting lipid removal from membranes or transfer between proteins. It has been shown that saposins, Niemann-Pick type C2 (NPC2) protein, GM2 activator protein, and CD1e can assist lipid binding to CD1d [47,48,49,50,51,52,53]. Saposins are important for endogenous and exogenous lipid removal and loading into mouse and human CD1d, both in the steady-state and during infection [47,48,49,50]. They perform lipid exchange, meaning that saposins are not capable of removing lipids from CD1d if they are not being replaced by another lipid. GM2 activator protein is able to remove mouse CD1d bound-lipids [48] and NPC2 protein can load lipids into mouse CD1d molecules [51]. Whether this is true for human cells has never been addressed. Finally, CD1e, which is present in human but not in mouse cells, promotes the loading and unloading of lipids on CD1d and on group I CD1 molecules [52,53]. This suggests that CD1e tunes the CD1d-mediated immune response by selecting ligands to load on CD1d molecules, consequently influencing presentation to both iNKT and type II NKT cells [53].

The lysosome is also a meeting point for CD1d molecules and lipid antigens. This is especially important for mouse CD1d molecules, which are known to localize mainly to the lysosome. In contrast, CD1d in humans is mostly targeted to late endosomes rather than lysosomes. Either way, both compartments carry lipids with long saturated tails, which may match the CD1d binding groove [27]. The loading of lipids in CD1d molecules is also facilitated by the low pH of the lysosome, which allows relaxation of the CD1d structure and consequently enables binding and dissociation of lipids [54]. Interestingly, in mice, type II NKT cells (identified as CD1d-restricted T cells that do not express Vα14 or NK1.1) do not depend on lysosomal localization of CD1d for activation [55]. Studies with tail-truncated mouse or human CD1d (not able to reach lysosomes or late endocytic compartments) showed how CD1d trafficking is important for lipid antigen presentation and iNKT cell development [50,56,57].

More recently, autophagy, a process regulated by the lysosome, was shown to be important for the development of iNKT cells [58,59], once again reinforcing the importance of the lysosome for NKT cell biology.

## 3. Lysosomal Storage and NKT Cells

### 3.1. NKT Cells in LSDs

The study of NKT cells in LSDs was centered on iNKT cells for a long time. Table 2 summarizes the previous studies analyzing NKT cells in animal models of LSDs. The first report describing iNKT cell alterations in LSDs was published in 2001 [41]. In this paper, preliminary data suggested that iNKT cells were reduced by approximately 50% in Fabry disease mice when compared to wild-type mice [41]. A few years later, a reduction in iNKT cell number was also described in a mouse model of Sandhoff disease [43]. Initially, these defects were thought to be disease-specific. However, in the following years, other papers were published describing iNKT cell alterations in GM1 gangliosidosis [60,61], Niemann-Pick type C1 (NPC1) [61,62], NPC2 [51,60], or Tay-Sachs [61] diseases, and confirming the alterations in Fabry [61,63,64,65] and Sandhoff diseases [61]. It was believed that lysosomal dysfunction per se, independently of the type of substrate stored, had a negative impact on the number of iNKT cells. However, a few years later, a report described normal iNKT cell numbers in mouse models of metachromatic leukodystrophy, mucopolysaccharidosis type I, and Krabbe disease [66]. These results raised the hypothesis that the iNKT cell defects encountered in LSDs were not merely caused by lysosomal storage, but related to the accumulation/absence of a specific group of lipids/enzymes. Recently, a decrease in the frequency of iNKT cells in a mouse model of Gaucher disease was reported [13]. In Table 3, studies on NKT in LSDs patients are listed. Earlier studies in Gaucher and Fabry disease patient, analyzed “iNKT-like” cells by the use of an antibody against Vα24 TCR α-chain, and no differences were observed between patients and controls [67,68,69]. Subsequent studies determined the frequency of iNKT cells in the peripheral blood of Fabry, Gaucher, and NPC disease patients, and no alterations in iNKT cell frequency were detected when compared with control subjects [13,70,71]. This can be attributed to the different trafficking pathways between human and mouse CD1d. While mouse CD1d is mainly found in the lysosomes, human CD1d is often found in late endosomes [14,72]. Thus, it is reasonable to suggest that mouse iNKT cells may be more affected by lysosomal dysfunction. Another possibility is related to the analysis of distinct tissues. In mouse models of LSDs, iNKT cells were analyzed in the thymus, spleen, and liver, whereas in patients, iNKT cells were collected from the blood. Blood contains a small number of iNKT cells and it was previously shown that the frequency of iNKT cells in blood do not correlate with the percentage of thymic iNKT cells [73]. Despite the absence of alterations in the frequency of iNKT cells in LSDs patients, the analysis of iNKT cell subsets revealed disease-specific alterations. While the iNKT cell population from Fabry disease patients has a decreased percentage of cells expressing CD4 with a concomitant increase of CD4− iNKT cells, iNKT cell subsets are unaffected in NPC patients [70,71]. Curiously, mouse models of both Fabry and NPC diseases display a reduction in iNKT cells expressing CD4 [60,64]. In the case of Fabry disease, in both mice and patients, these phenotypic alterations were accompanied by functional alterations accessory to a pro-inflammatory status that has been described in these patients [71,74]. Thus, iNKT cells might play a role in vascular inflammation leading to endothelial cell dysfunction, a feature of Fabry disease.

The analysis of type II NKT cells is complicated by the lack of specific markers that identify these cells. Nevertheless, a recent study used CD1d tetramers to identify glucosylceramide and glucosylsphingosine-specific type II NKT cells in Gaucher disease mice and patients [13]. They found that glucosylsphingosine-specific type II NKT cells were increased in both mice and patients when compared to control subjects [13]. Importantly, in Gaucher disease patients, the frequency of these glucosylsphingosine-specific type II NKT cells is positively correlated with the activity of chitotriosidase in serum, an established biomarker of disease severity [13,75]. This, together with the fact that these cells strongly communicate with B cells, suggests a possible implication of type II NKT cells in the pathology of Gaucher disease, and more specifically in the development of B cell malignancies.

### 3.2. Proposed Mechanisms Behind NKT Cell Alterations

**Figure 1:**
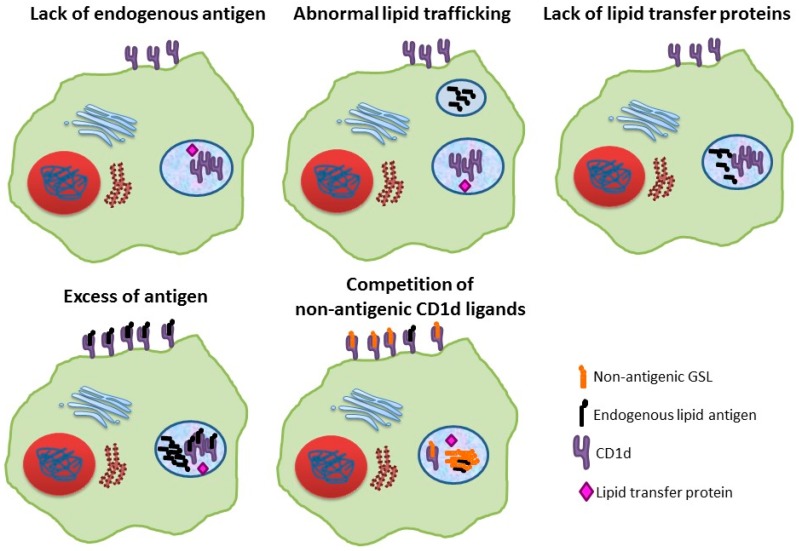
Proposed mechanisms, occurring in the antigen-presenting cell, that can explain invariant Natural Killer T (iNKT) cell defects in lysosomal storage diseases (LSDs). GSL, glycosphingolipid.

Although a clear relationship between lysosomal dysfunction and alterations in the iNKT cell compartment was established more than 10 years ago, the elucidation of the mechanisms behind these alterations continues until today. Importantly, all explanations are based on events occurring at the antigen presenting cell, which lead to an alteration in the amount of CD1d molecules bound to endogenous antigens at the cell surface and consequently to an impaired iNKT cell selection or activation (Figure 1).

The first explanation was proposed together with the first description of a reduction in iNKT cell numbers in Fabry disease [41]. In this study, α-galactosidase A was shown to be necessary for the processing of a synthetic glycolipid, which upon galactose removal gains antigenic activity [41]. Even though they could not exclude other mechanisms, the lack of correct antigen processing was proposed as the mechanism behind iNKT cell reduction. By analyzing lipid antigen presentation in the Sandhoff mouse model, β-hexosaminidase was identified as being essential for the generation of isoglobotriosylceramide (iGb3), an endogenous iNKT cells antigen [43]. Therefore, in the absence of this enzyme, there is no available antigen for thymic selection of iNKT cells, leading to a reduction in their numbers. Later, a putative accumulation of iGb3 in the Fabry disease mouse model has been suggested to be the link to the overstimulation and decreased number of iNKT cells in these mice [63]. However, the accumulation of iGb3 was not addressed directly. The presence of iGb3 in the thymus was controversial, and in 2012 it was shown that, in Fabry disease mice, the storage of globotriaosylceramide (Gb3) is responsible for the reduction of both iNKT cell numbers and antigen presentation [65]. Indeed, it is now known that different antigens can select iNKT cells [46,76]. Sagiv and colleagues proposed an explanation for the iNKT cell reduction observed in NPC1-deficient mouse model [62]. They demonstrated that the abnormal lipid trafficking existent in NPC1 cells prevented the co-localization of iNKT cell antigens with CD1d molecules, thus preventing the loading of antigens onto CD1d molecules [62]. Soon after, the NPC2 protein, defective in NPC2 disease, was shown to be fundamental for the loading of lipid antigens in CD1d, working as a LTP. This justified the strong reduction of iNKT cells observed in NPC2-deficient mice [51,60]. At this point, the proposed mechanisms behind iNKT cell alterations were related to the lack of antigen presentation and consequent defective selection of iNKT cells in the thymus. This idea was then challenged by the discovery that the enzyme α-galactosidase A, defective in Fabry disease, has a role in the degradation of endogenous antigens [46], suggesting that the reduction in iNKT cell numbers could be related not to a lack of selection, but instead , due to the death caused by an overstimulation potentiated by an excess of endogenous antigen. This notion was further supported by the identification of signals of chronic activation in residual iNKT cells from Fabry disease mice [63]. It is known that several GSLs have the capacity to bind to CD1d molecules [14], however, the impact of this binding in NKT cell activation is not clear. One of the explanations proposed in 2006 by Gadola and co-workers was that the high amounts of lipids accumulating in the lysosomes could easily out-compete the endogenous antigens for CD1d binding, thus leading to iNKT cell defects [61]. This hypothesis was recently proven for the GSL Gb3, which accumulates in high amounts in Fabry disease. Gb3 was identified as a CD1d ligand capable of inhibiting iNKT cell activation by competing with endogenous antigens for CD1d binding [77].

### 3.3. The Effect of Enzyme Replacement Therapy on NKT Cells

Enzyme replacement therapy (ERT) is available for some LSDs, namely Gaucher disease, Fabry disease, Pompe disease, mucopolysaccharidosis (MPS)-I, MPS-II, MPS-Iva, and MPS VI [78]. ERT consists of the infusion of a recombinant enzyme that is targeted to the lysosomes, thus recovering the function of the defective enzyme. For some LSDs, this treatment option successfully ameliorates disease pathology. Due to the properties of the blood-brain barrier, ERT is not applicable to treat pathologies of the central nervous system that are characteristic to some LSDs. In Gaucher disease, the first disease for which ERT was available, ERT is very successful, correcting hepatosplenomegaly and thrombocytopenia and reducing bone complications [79,80]. In Fabry disease, clinical trials have shown an improvement in pain and in cardiac disease [81]. However, little is known about the effect of ERT on NKT cells.

In the Fabry disease mouse model, ERT was shown to partially prevent the decrease in the number of splenic iNKT cells [64]. However, it was not capable of correcting the defect already existent at the beginning of the ERT [64]. When the number of iNKT cells was compared between patients receiving ERT and not receiving ERT, no significant alterations were found [69]. Similarly, a longitudinal analysis starting four months after the beginning of the ERT and along a total of 24 months (with a four month periodicity) revealed no significant alterations in the frequency of iNKT cells or iNKT CD4/CD8 subsets [71]. In Gaucher disease patients, the levels of CD4+ and CD8+ T cells expressing the TCR chain Vα24—a population that includes, but is not limited to, iNKT cells—were similar between untreated and treated patients, suggesting that ERT does not have an influence in iNKT cell frequency or phenotype [67]. Contrarily, ERT seems to successfully reduce the increase of glucosylsphingosine-specific type II NKT cells observed in Gaucher disease patients [13]. This might be related to different antigen presentation requirements between iNKT and type II NKT cells. The type II NKT cells analyzed in this study are specific to glucosylsphingosine. Therefore, it is expected that by reducing the amount of antigen available through ERT, the glucosylsphingosine-specific type II NKT cells would decrease. iNKT cells, on the contrary, can recognize different antigens, and the defects observed in LSDs may be explained by distinct mechanisms that are not be overcome by ERT. Additional studies, targeting the characterization of iNKT cells in LSDs before and at different times of ERT and analyzing the effect of ERT on the different proposed mechanisms behind iNKT cell alterations, would be important in order to clarify this subject.

## 4. Conclusions

A tight relationship between NKT cells and LSDs is now established. The lysosomal impairment observed in LSDs is sufficient to cause alterations in this subset of T cells. However, at this point, the contribution of NKT cell defects to LSDs pathology is not completely understood. Nevertheless, NKT cells are known to play a pivotal role in autoimmunity, infections, and cancer, conditions for which some patients with LSDs present increased risk [82,83,84]. It is important to continue studying NKT cells in the context of LSDs. Ideally, these studies should be performed in human samples because of the differences in NKT cell biology between humans and mice. Another important task would be to correlate NKT cell alterations with clinical parameters in patients in order to identify new biomarkers. Finally, the study of NKT cells in LSDs is crucial to unveil the importance of the lysosome and of different lipids in NKT cell biology, thereby opening new ways to modulate NKT cell responses.

## Figures and Tables

**Table 1:** Main differences between iNKT and type II NKT cells. NKT, Natural Killer T; iNKT, invariant NKT; TCR, T cell receptor; CD1d, cluster of differentiation 1 d.

Feature	iNKT Cells	Type II NKT Cells
TCR	Semi-invariant; Vα24Jα18 Vβ11 (humans) and Vα14Jα18 (mice)	Variable; αβ or γδ
Preferred Antigens	α-linked monohexosylceramides	Phospholipids; β-linked glycosphingolipids
Antigen Specificity	All cells recognize the same antigen	Different cells have different antigen specificities
Identification	CD1d tetramers loaded with specific antigen; Antibodies against semi-invariant TCR	CD1d tetramers loaded with specific antigen
Whole Population Identified?	Yes	No

**Table 2:** NKT cells in Lysosomal Storage Diseases mouse models.

Disease	Protein Defect	iNKT Cells	Type II NKT Cells	iNKT Cell Subsets
Fabry	α-galactosidase A	↓ [41,61,63,64,65]	ND	↓ CD4+ iNKT cells [64]
NPC1	NPC1	↓ [61,62]	ND	ND
NPC2	NPC2	↓ [51,60]	ND	↓ CD4+ iNKT cells [60]
Sandhoff	β-hexosaminidase A/B	↓ [43,61]	ND	ND
	β subunit			
Tay-Sachs	β-hexosaminidase A	↓ [61]	ND	ND
	α subunit			
GM1 gangliosidosis	β-galactosidase	↓ [60,61]	ND	ND
Metachromatic leukodystrophy	Arylsulphatase A	= [66]	ND	ND
MPS I	α-L-iduronidase	= [66]	ND	ND
Krabbe	β-galactosylceramidase	= [66]	ND	ND
Gaucher	β-glucosidase (Glucocerebrosidase)	↓ [13]	↑ (glucosylsphingosine-specific) [13]	ND

=, normal; ↑, increase; ↓, reduction; ND, not done; MPS I, Mucopolysaccharidosis type I; NPC, Niemann-Pick type C.

**Table 3:** NKT cells in Lysosomal Storage Diseases (LSDs) patients.

Disease	Protein Defect	iNKT Cells	Type II NKT Cells	iNKT Cell Subsets
Gaucher	β-glucosidase (Glucocerebrosidase)	= [13]	↑ (glucosylsphingosine-specific) [13]	ND
Fabry	α-galactosidase A	= [71]	ND	↓ CD4+ iNKT cells [71]
NPC	NPC1	= [70]	ND	No alterations in CD4+ iNKT cells [70]

=, normal; ↑, increase; ↓, reduction; ND, not done; NPC, Niemann-Pick type C.

## References

[1] De Duve C., Pressman B.C., Gianetto R., Wattiaux R., Appelmans F. (1955). Tissue fractionation studies. 6. Intracellular distribution patterns of enzymes in rat-liver tissue. Biochem. J..

[2] Segatori L. (2014). Impairment of homeostasis in lysosomal storage disorders. IUBMB Life.

[3] Parenti G., Andria G., Ballabio A. (2015). Lysosomal storage diseases: From pathophysiology to therapy. Ann. Rev. Med..

[4] Alroy J., Garganta C., Wiederschain G. (2014). Secondary biochemical and morphological consequences in lysosomal storage diseases. Biochem. Moscow.

[5] Mori L., Lepore M., de Libero G. (2016). The immunology of CD1- and MR1-restricted T cells. Ann. Rev. Immunol..

[6] Godfrey D.I., MacDonald H.R., Kronenberg M., Smyth M.J., van Kaer L. (2004). NKT cells: What’s in a name?. Nat. Rev. Immunol..

[7] Kumar V., Delovitch T.L. (2014). Different subsets of natural killer T cells may vary in their roles in health and disease. Immunology.

[8] Kohlgruber A.C., Donado C.A., LaMarche N.M., Brenner M.B., Brennan P.J. (2016). Activation strategies for invariant natural killer T cells. Immunogenetics.

[9] Rhost S., Sedimbi S., Kadri N., Cardell S.L. (2012). Immunomodulatory type II natural killer T lymphocytes in health and disease. Scand. J. Immunol..

[10] Marrero I., Ware R., Kumar V. (2015). Type II NKT cells in inflammation, autoimmunity, microbial immunity, and cancer. Front. Immunol..

[11] Rhost S., Lofbom L., Rynmark B.-M., Pei B., Mansson J.-E., Teneberg S., Blomqvist M., Cardell S.L. (2012). Identification of novel glycolipid ligands activating a sulfatide-reactive, CD1d-restricted, type II natural killer T lymphocyte. Eur. J. Immunol..

[12] Maricic I., Girardi E., Zajonc D.M., Kumar V. (2014). Recognition of lysophosphatidylcholine by type II NKT cells and protection from an inflammatory liver disease. J. Immunol..

[13] Nair S., Boddupalli C.S., Verma R., Liu J., Ruhua Y., Pastores G.M., Mistry P., Dhodapkar M.V. (2014). Type II NKT-TFH cells against gaucher lipids regulate B cell immunity and inflammation. Blood.

[14] Pereira C.S., Macedo M.F. (2016). CD1-restricted T cells at the crossroad of innate and adaptive immunity. J. Immunol. Res..

[15] Jahng A., Maricic I., Aguilera C., Cardell S., Halder R.C., Kumar V. (2004). Prevention of autoimmunity by targeting a distinct, noninvariant CD1d-reactive T cell population reactive to sulfatide. J. Exp. Med..

[16] Arrenberg P., Halder R., Dai Y., Maricic I., Kumar V. (2010). Oligoclonality and innate-like features in the TCR repertoire of type II NKT cells reactive to a β-linked self-glycolipid. Proc. Natl. Acad. Sci. USA.

[17] Montoya C.J., Pollard D., Martinson J., Kumari K., Wasserfall C., Mulder C.B., Rugeles M.T., Atkinson M.A., Landay A.L., Wilson S.B. (2007). Characterization of human invariant natural killer t subsets in health and disease using a novel invariant natural killer T cell-clonotypic monoclonal antibody, 6b11. Immunology.

[18] Gumperz J.E., Miyake S., Yamamura T., Brenner M.B. (2002). Functionally distinct subsets of CD1d-restricted natural killer T cells revealed by CD1d tetramer staining. J. Exp. Med..

[19] Berzins S.P., Smyth M.J., Baxter A.G. (2011). Presumed guilty: Natural killer T cell defects and human disease. Nat. Rev. Immunol..

[20] Lynch L., O’Shea D., Winter D.C., Geoghegan J., Doherty D.G., O’Farrelly C. (2009). Invariant NKT cells and CD1d+ cells amass in human omentum and are depleted in patients with cancer and obesity. Eur. J. Immunol..

[21] Lynch L., Nowak M., Varghese B., Clark J., Hogan A.E., Toxavidis V., Balk S.P., O’Shea D., O’Farrelly C., Exley M.A. (2012). Adipose tissue invariant NKT cells protect against diet-induced obesity and metabolic disorder through regulatory cytokine production. Immunity.

[22] Schipper H.S., Rakhshandehroo M., van de Graaf S.F., Venken K., Koppen A., Stienstra R., Prop S., Meerding J., Hamers N., Besra G. (2012). Natural killer T cells in adipose tissue prevent insulin resistance. J. Clin. Investig..

[23] Wu L., Parekh V.V., Gabriel C.L., Bracy D.P., Marks-Shulman P.A., Tamboli R.A., Kim S., Mendez-Fernandez Y.V., Besra G.S., Lomenick J.P. (2012). Activation of invariant natural killer T cells by lipid excess promotes tissue inflammation, insulin resistance, and hepatic steatosis in obese mice. Proc. Natl. Acad. Sci. USA.

[24] Lynch L., Michelet X., Zhang S., Brennan P.J., Moseman A., Lester C., Besra G., Vomhof-Dekrey E.E., Tighe M., Koay H.F. (2015). Regulatory inkt cells lack expression of the transcription factor PLZF and control the homeostasis of T_reg_ cells and macrophages in adipose tissue. Nat. Immunol..

[25] Lynch L., Hogan A.E., Duquette D., Lester C., Banks A., LeClair K., Cohen D.E., Ghosh A., Lu B., Corrigan M. (2016). iNKT cells induce FGF21 for thermogenesis and are required for maximal weight loss in GLP1 therapy. Cell Metab..

[26] Satoh M., Hoshino M., Fujita K., Iizuka M., Fujii S., Clingan C.S., van Kaer L., Iwabuchi K. (2016). Adipocyte-specific CD1d-deficiency mitigates diet-induced obesity and insulin resistance in mice. Sci. Rep..

[27] Salio M., Silk J.D., Jones E.Y., Cerundolo V. (2014). Biology of CD1- and MR1-restricted T cells. Ann. Rev. Immunol..

[28] Godfrey D.I., Stankovic S., Baxter A.G. (2010). Raising the NKT cell family. Nat. Immunol..

[29] Lee P.T., Benlagha K., Teyton L., Bendelac A. (2002). Distinct functional lineages of human Vα24 natural killer T cells. J. Exp. Med..

[30] Takahashi T., Chiba S., Nieda M., Azuma T., Ishihara S., Shibata Y., Juji T., Hirai H. (2002). Cutting edge: Analysis of human Vα24+CD8+ NK T cells activated by α-galactosylceramide-pulsed monocyte-derived dendritic cells. J. Immunol..

[31] O’Reilly V., Zeng S.G., Bricard G., Atzberger A., Hogan A.E., Jackson J., Feighery C., Porcelli S.A., Doherty D.G. (2011). Distinct and overlapping effector functions of expanded human CD4^+^, CD8ALPHA^+^ and CD4^−^CD8Α^−^ invariant natural killer T cells. PLoS ONE.

[32] Moreira-Teixeira L., Resende M., Coffre M., Devergne O., Herbeuval J.P., Hermine O., Schneider E., Rogge L., Ruemmele F.M., Dy M. (2011). Proinflammatory environment dictates the IL-17-producing capacity of human invariant NKT cells. J. Immunol..

[33] Monteiro M., Almeida C.F., Agua-Doce A., Graca L. (2013). Induced IL-17-producing invariant NKT cells require activation in presence of TGF-β and IL-1β. J. Immunol..

[34] Monteiro M., Agua-Doce A., Almeida C.F., Fonseca-Pereira D., Veiga-Fernandes H., Graca L. (2015). IL-9 expression by invariant NKT cells is not imprinted during thymic development. J. Immunol..

[35] Cardell S., Tangri S., Chan S., Kronenberg M., Benoist C., Mathis D. (1995). CD1-restricted CD4+ T cells in major histocompatibility complex class II-deficient mice. J. Exp. Med..

[36] Park S.H., Weiss A., Benlagha K., Kyin T., Teyton L., Bendelac A. (2001). The mouse cd1d-restricted repertoire is dominated by a few autoreactive T cell receptor families. J. Exp. Med..

[37] Exley M.A., Tahir S.M., Cheng O., Shaulov A., Joyce R., Avigan D., Sackstein R., Balk S.P. (2001). A major fraction of human bone marrow lymphocytes are Th2-like CD1d-reactive T cells that can suppress mixed lymphocyte responses. J. Immunol..

[38] Exley M.A., He Q., Cheng O., Wang R.J., Cheney C.P., Balk S.P., Koziel M.J. (2002). Cutting edge: Compartmentalization of Th1-like noninvariant CD1d-reactive T cells in hepatitis c virus-infected liver. J. Immunol..

[39] Chang D.H., Deng H., Matthews P., Krasovsky J., Ragupathi G., Spisek R., Mazumder A., Vesole D.H., Jagannath S., Dhodapkar M.V. (2008). Inflammation-associated lysophospholipids as ligands for CD1D-restricted T cells in human cancer. Blood.

[40] Bai L., Picard D., Anderson B., Chaudhary V., Luoma A., Jabri B., Adams E.J., Savage P.B., Bendelac A. (2012). The majority of CD1D-sulfatide-specific T cells in human blood use a semiinvariant VΔ1 TCR. Eur. J. Immunol..

[41] Prigozy T.I., Naidenko O., Qasba P., Elewaut D., Brossay L., Khurana A., Natori T., Koezuka Y., Kulkarni A., Kronenberg M. (2001). Glycolipid antigen processing for presentation by CD1d molecules. Science.

[42] Kinjo Y., Illarionov P., Vela J.L., Pei B., Girardi E., Li X., Li Y., Imamura M., Kaneko Y., Okawara A. (2011). Invariant natural killer T cells recognize glycolipids from pathogenic gram-positive bacteria. Nat. Immunol..

[43] Zhou D., Mattner J., Cantu C., Schrantz N., Yin N., Gao Y., Sagiv Y., Hudspeth K., Wu Y.P., Yamashita T. (2004). Lysosomal glycosphingolipid recognition by NKT cells. Science.

[44] Fox L.M., Cox D.G., Lockridge J.L., Wang X., Chen X., Scharf L., Trott D.L., Ndonye R.M., Veerapen N., Besra G.S. (2009). Recognition of lyso-phospholipids by human natural killer T lymphocytes. PLoS Biol..

[45] Paduraru C., Bezbradica J.S., Kunte A., Kelly R., Shayman J.A., Veerapen N., Cox L.R., Besra G.S., Cresswell P. (2013). Role for lysosomal phospholipase A2 in iNKT cell-mediated CD1d recognition. Proc. Natl. Acad. Sci. USA.

[46] Kain L., Webb B., Anderson B.L., Deng S., Holt M., Costanzo A., Zhao M., Self K., Teyton A., Everett C. (2014). The identification of the endogenous ligands of natural killer T cells reveals the presence of mammalian α-linked glycosylceramides. Immunity.

[47] Kang S.J., Cresswell P. (2004). Saposins facilitate CD1D-restricted presentation of an exogenous lipid antigen to T cells. Nat. Immunol..

[48] Zhou D., Cantu C., Sagiv Y., Schrantz N., Kulkarni A.B., Qi X., Mahuran D.J., Morales C.R., Grabowski G.A., Benlagha K. (2004). Editing of CD1d-bound lipid antigens by endosomal lipid transfer proteins. Science.

[49] Yuan W., Qi X., Tsang P., Kang S.J., Illarionov P.A., Besra G.S., Gumperz J., Cresswell P. (2007). Saposin b is the dominant saposin that facilitates lipid binding to human CD1d molecules. Proc. Natl. Acad. Sci. USA.

[50] Salio M., Ghadbane H., Dushek O., Shepherd D., Cypen J., Gileadi U., Aichinger M.C., Napolitani G., Qi X., van der Merwe P.A. (2013). Saposins modulate human invariant natural killer T cells self-reactivity and facilitate lipid exchange with CD1d molecules during antigen presentation. Proc. Natl. Acad. Sci. USA.

[51] Schrantz N., Sagiv Y., Liu Y., Savage P.B., Bendelac A., Teyton L. (2007). The niemann-pick type C2 protein loads isoglobotrihexosylceramide onto CD1d molecules and contributes to the thymic selection of NKT cells. J. Exp. Med..

[52] de la Salle H., Mariotti S., Angenieux C., Gilleron M., Garcia-Alles L.F., Malm D., Berg T., Paoletti S., Maitre B., Mourey L. (2005). Assistance of microbial glycolipid antigen processing by CD1e. Science.

[53] Facciotti F., Cavallari M., Angenieux C., Garcia-Alles L.F., Signorino-Gelo F., Angman L., Gilleron M., Prandi J., Puzo G., Panza L. (2011). Fine tuning by human CD1e of lipid-specific immune responses. Proc. Natl. Acad. Sci. USA.

[54] De Libero G., Mori L. (2012). Novel insights into lipid antigen presentation. Trends Immunol..

[55] Chiu Y.H., Jayawardena J., Weiss A., Lee D., Park S.H., Dautry-Varsat A., Bendelac A. (1999). Distinct subsets of cd1d-restricted T cells recognize self-antigens loaded in different cellular compartments. J. Exp. Med..

[56] Chiu Y.H., Park S.H., Benlagha K., Forestier C., Jayawardena-Wolf J., Savage P.B., Teyton L., Bendelac A. (2002). Multiple defects in antigen presentation and T cell development by mice expressing cytoplasmic tail-truncated CD1d. Nat. Immunol..

[57] Chen X., Wang X., Keaton J.M., Reddington F., Illarionov P.A., Besra G.S., Gumperz J.E. (2007). Distinct endosomal trafficking requirements for presentation of autoantigens and exogenous lipids by human CD1d molecules. J. Immunol..

[58] Salio M., Puleston D.J., Mathan T.S., Shepherd D., Stranks A.J., Adamopoulou E., Veerapen N., Besra G.S., Hollander G.A., Simon A.K. (2014). Essential role for autophagy during invariant NKT cell development. Proc. Natl. Acad. Sci. USA.

[59] Pei B., Zhao M., Miller B.C., Vela J.L., Bruinsma M.W., Virgin H.W., Kronenberg M. (2015). Invariant nkt cells require autophagy to coordinate proliferation and survival signals during differentiation. J. Immunol..

[60] Schumann J., Facciotti F., Panza L., Michieletti M., Compostella F., Collmann A., Mori L., de Libero G. (2007). Differential alteration of lipid antigen presentation to NKT cells due to imbalances in lipid metabolism. Eur. J. Immunol..

[61] Gadola S.D., Silk J.D., Jeans A., Illarionov P.A., Salio M., Besra G.S., Dwek R., Butters T.D., Platt F.M., Cerundolo V. (2006). Impaired selection of invariant natural killer T cells in diverse mouse models of glycosphingolipid lysosomal storage diseases. J. Exp. Med..

[62] Sagiv Y., Hudspeth K., Mattner J., Schrantz N., Stern R.K., Zhou D., Savage P.B., Teyton L., Bendelac A. (2006). Cutting edge: Impaired glycosphingolipid trafficking and NKT cell development in mice lacking niemann-pick type c1 protein. J. Immunol..

[63] Darmoise A., Teneberg S., Bouzonville L., Brady R.O., Beck M., Kaufmann S.H., Winau F. (2010). Lysosomal α-galactosidase controls the generation of self lipid antigens for natural killer T cells. Immunity.

[64] Macedo M.F., Quinta R., Pereira C.S., Sa Miranda M.C. (2012). Enzyme replacement therapy partially prevents invariant natural killer T cell deficiency in the fabry disease mouse model. Mol. Genet. Metab..

[65] Porubsky S., Speak A.O., Salio M., Jennemann R., Bonrouhi M., Zafarulla R., Singh Y., Dyson J., Luckow B., Lehuen A. (2012). Globosides but not isoglobosides can impact the development of invariant NKT cells and their interaction with dendritic cells. J. Immunol..

[66] Plati T., Visigalli I., Capotondo A., Buono M., Naldini L., Cosma M.P., Biffi A. (2009). Development and maturation of invariant NKT cells in the presence of lysosomal engulfment. Eur. J. Immunol..

[67] Balreira A., Lacerda L., Miranda C.S., Arosa F.A. (2005). Evidence for a link between sphingolipid metabolism and expression of CD1d and MHC-class II: Monocytes from gaucher disease patients as a model. Br. J. Haematol..

[68] Balreira A., Macedo M.F., Girao C., Rodrigues L.G., Oliveira J.P., Sa Miranda M.C., Arosa F.A. (2008). Anomalies in conventional T and invariant natural killer T-cell populations in fabry mice but not in fabry patients. Br. J. Haematol..

[69] Rozenfeld P., Agriello E., de Francesco N., Martinez P., Fossati C. (2009). Leukocyte perturbation associated with fabry disease. J. Inherit. Metab. Dis..

[70] Speak A.O., Platt N., Salio M., te Vruchte D., Smith D.A., Shepherd D., Veerapen N., Besra G.S., Yanjanin N.M., Simmons L. (2012). Invariant natural killer T cells are not affected by lysosomal storage in patients with niemann-pick disease type c. Eur. J. Immunol..

[71] Pereira C.S., Azevedo O., Luz Maia M., Dias A.F., Sa-Miranda C., Fatima Macedo M. (2013). Invariant natural killer T cells are phenotypically and functionally altered in fabry disease. Mol. Genet. Metab..

[72] Vartabedian V.F., Savage P.B., Teyton L. (2016). The processing and presentation of lipids and glycolipids to the immune system. Immunol. Rev..

[73] Berzins S.P., Cochrane A.D., Pellicci D.G., Smyth M.J., Godfrey D.I. (2005). Limited correlation between human thymus and blood NKT cell content revealed by an ontogeny study of paired tissue samples. Eur. J. Immunol..

[74] Biancini G.B., Vanzin C.S., Rodrigues D.B., Deon M., Ribas G.S., Barschak A.G., Manfredini V., Netto C.B., Jardim L.B., Giugliani R. (2012). Globotriaosylceramide is correlated with oxidative stress and inflammation in fabry patients treated with enzyme replacement therapy. Biochim. Biophys. Acta.

[75] Hollak C.E., van Weely S., van Oers M.H., Aerts J.M. (1994). Marked elevation of plasma chitotriosidase activity. A novel hallmark of gaucher disease. J. Clin.Investig..

[76] Facciotti F., Ramanjaneyulu G.S., Lepore M., Sansano S., Cavallari M., Kistowska M., Forss-Petter S., Ni G., Colone A., Singhal A. (2012). Peroxisome-derived lipids are self antigens that stimulate invariant natural killer T cells in the thymus. Nat. Immunol..

[77] Pereira C.S., Sa-Miranda C., De Libero G., Mori L., Macedo M.F. (2016). Globotriaosylceramide inhibits iNKT-cell activation in a CD1d-dependent manner. Eur. J. Immunol..

[78] Pastores G.M., Hughes D.A. (2015). Non-neuronopathic lysosomal storage disorders: Disease spectrum and treatments. Best Pract. Res. Clin. Endocrinol. Metab..

[79] Bennett L.L., Mohan D. (2013). Gaucher disease and its treatment options. Ann. Pharmacother..

[80] Grabowski G.A.P., Edwin H., Valle D., Beaudet A.L., Vogelstein B., Kinzler K.W., Antonarakis S.E., Ballabio A., Gibson K.M., Mitchell G. (2013). Kolodny Gaucher Disease. The Online Metabolic and Molecular Bases of Inherited Disease.

[81] Hollak C.E., Weinreb N.J. (2015). The attenuated/late onset lysosomal storage disorders: Therapeutic goals and indications for enzyme replacement treatment in gaucher and fabry disease. Best Pract. Res. Clin. Endocrinol. Metab..

[82] Choy F.Y., Campbell T.N. (2011). Gaucher disease and cancer: Concept and controversy. Int. J. Cell Biol..

[83] Vitner E.B., Platt F.M., Futerman A.H. (2010). Common and uncommon pathogenic cascades in lysosomal storage diseases. J. Biol. Chem..

[84] Killedar S., Dirosario J., Divers E., Popovich P.G., McCarty D.M., Fu H. (2010). Mucopolysaccharidosis IIIb, a lysosomal storage disease, triggers a pathogenic CNS autoimmune response. J. Neuroinflamm..

